# Influence of organic versus inorganic dietary selenium supplementation on the concentration of selenium in colostrum, milk and blood of beef cows

**DOI:** 10.1186/1751-0147-50-43

**Published:** 2008-11-03

**Authors:** Petr Slavik, Josef Illek, Michal Brix, Jaroslava Hlavicova, Radko Rajmon, Frantisek Jilek

**Affiliations:** 1 Department of Veterinary Sciences, Czech University of Life Sciences Prague, Kamycka 129, Prague 6, CZ165 21, Czech Republic; 2 Clinic of Ruminant Diseases, University of Veterinary and Pharmaceutical Sciences Brno, Palackeho 1/3 Brno, CZ612 42, Czech Republic; 3 Private Veterinary Practice, Blumentalska 22, Bratislava, SK811 07, Slovakia

## Abstract

**Background:**

Selenium (Se) is important for the postnatal development of the calf. In the first weeks of life, milk is the only source of Se for the calf and insufficient level of Se in the milk may lead to Se deficiency. Maternal Se supplementation is used to prevent this.

We investigated the effect of dietary Se-enriched yeast (SY) or sodium selenite (SS) supplements on selected blood parameters and on Se concentrations in the blood, colostrum, and milk of Se-deficient Charolais cows.

**Methods:**

Cows in late pregnancy received a mineral premix with Se (SS or SY, 50 mg Se per kg premix) or without Se (control – C). Supplementation was initiated 6 weeks before expected calving. Blood and colostrum samples were taken from the cows that had just calved (Colostral period). Additional samples were taken around 2 weeks (milk) and 5 weeks (milk and blood) after calving corresponding to Se supplementation for 6 and 12 weeks, respectively (Lactation period) for Se, biochemical and haematological analyses.

**Results:**

Colostral period. Se concentrations in whole blood and colostrum on day 1 *post partum *and in colostrum on day 3 *post partum *were 93.0, 72.9, and 47.5 μg/L in the SY group; 68.0, 56.0 and 18.8 μg/L in the SS group; and 35.1, 27.3 and 10.5 μg/L in the C group, respectively. Differences among all the groups were significant (*P *< 0.01) at each sampling, just as the colostrum Se content decreases were from day 1 to day 3 in each group. The relatively smallest decrease in colostrum Se concentration was found in the SY group (*P *< 0.01).

Lactation period. The mean Se concentrations in milk in weeks 6 and 12 of supplementation were 20.4 and 19.6 μg/L in the SY group, 8.3 and 11.9 μg/L in the SS group, and 6.9 and 6.6 μg/L in the C group, respectively. The values only differed significantly in the SS group (*P *< 0.05). The Se concentrations in the blood were similar to those of cows examined on the day of calving. The levels of glutathione peroxidase (GSH-Px) activity were 364.70, 283.82 and 187.46 μkat/L in the SY, SS, and C groups, respectively. This was the only significantly variable biochemical and haematological parameter.

**Conclusion:**

Se-enriched yeast was much more effective than sodium selenite in increasing the concentration of Se in the blood, colostrum and milk, as well as the GSH-Px activity.

## Background

Selenium (Se) is extremely important for the proper postnatal development of the calf. Selenium deficiency compromises growth, health and fertility [1]. During pregnancy, Se passes through the placental barrier, and even if the cow is moderately Se deficient, the calf receives a sufficient Se supply [2]. In the first weeks of life, milk is the only source of Se for the calf, but the Se content in the milk is rather low [3]. Therefore, calves from dams with insufficient Se supplementation may suffer from myodystrophic diseases [4] or other disorders related to Se deficiency.

It has been demonstrated that the Se content in colostrum is higher than in milk [5,6], but the dynamics of the Se concentration in colostrum and milk are unknown. Also, very variable Se concentrations in colostrum or milk and varying correlations between Se concentrations in blood and milk have been observed [5-10]. Besides being due to the nutritional level of Se, this may also reflect differences in milk yield and cattle breed among the studies. Beef cattle have been studied to a lesser extent than dairy cattle although beef calves are generally more dependent on the dams' milk than calves from dairy farms, which often receive milk substitutes.

The Se content in body fluids depends considerably on the Se intake from the diet. Dietary supplementation with Se-enriched yeast (SY) results in higher concentrations of Se in cows' milk than inorganic Se (SS) supplementation does [11]. Replacing inorganic Se with organic Se has been suggested as one way to increase Se intake in humans in areas with suboptimal Se status [12]. Studies performed mainly on dairy cattle have shown an increase of Se concentration in the milk of animals supplemented with SY as compared with those supplemented with SS. The increase has ranged from 34% [9] to 90% [11]. Although Se is an essential mineral, the effects of Se supplementation on haematological or biochemical parameters are considered minimal in cattle without significant Se deficiency [9,13]. Biochemical changes in Se deficient cattle include changed activity of the enzymes aspartate aminotransferase (AST), creatine kinase (CK), and glutathione peroxidase (GSH-Px) [14]. Also, significant differences in blood urea concentration have been observed after Se treatment [9].

GSH-Px activity is considered to be an indicator of long-term Se supply, as it depends on the erythrocyte life cycle [14]. However, it has been discussed how rapidly the GSH-Px activity reflects changes in the Se status. Also, the relationship between GSH-Px activity and the Se form in the diet (SS or SY) is not definite [15].

The objective of this study was to investigate 1) Se concentrations in colostrum and milk in a Se deficient beef cattle herd, 2) the effect of feeding premixes supplemented with SY or SS on Se concentrations in the blood, colostrum, and milk and on the dynamics of the Se concentration, and 3) the effect of providing different dietary Se sources on selected blood biochemical parameters in Se deficient animals.

## Methods

### Management, animals and feeding

The study was conducted in a Charolais herd in the Vysocina region, Czech Republic. The herd included about 350 head of cattle of which 180 were reproductive. The animals were located at several sites relatively close to each other. The winter diet consisted of grass silage and hay. Grazing was provided in early spring and late autumn. The calving period lasted from March through May.

### Experimental design

The study lasted three consecutive months (starting in March 2007) and included 120 late pregnant cows. There were 3 experimental groups of 40 cows each. Two groups (SY and SS) were fed the same mineral premix supplemented with 50 mg Se/kg premix. The SY group received an organic Se source (Se-enriched yeast Sel-Plex 50, Alltech, Nicholasville, KY) and the SS group received an inorganic Se supplement (sodium selenite) while the control group received a premix without Se supplementation. Se supplementation was initiated 6 weeks before the expected calving period.

All the cows received the same basic diet and had free access to a micro-pellet mineral feed (Ca 12%, P 6%, Na 11%, Mg 13%, Cu 1500 mg/kg, Mn 2100 mg/kg, Zn 1100 mg/kg, I 100 mg/kg, Co 40 mg/kg, vitamin A 1000000 I.U./kg, vitamin D 100000 I.U./kg, vitamin E 1000 mg/kg).

The Se status was determined at the beginning of the study by analyzing whole blood of 20 randomly selected cows.

Different experimental set-ups were used in the 2 study parts (colostrum and lactation periods, respectively). Sampling during the colostrum period was done on the day of calving and on day 3 *post partum*. As the cows calved continuously, the preceding period of Se supplementation varied, but was never less than 6 weeks. Sampling during the lactation period was done after a fixed period of 6 and 12 weeks Se supplementation. The period from calving to date of sampling varied because the time of Se supplementation was fixed and the animals calved continuously, but it was never less than 10 days. Data on individual cows is shown in additional files 1 and 2 (Additional file 1: Individual data on cows analysed for selenium content in colostrum; Additional file 2: Individual data on cows analysed for selenium content in milk).

The concentration of Se in the colostrum was determined twice from 6 randomly selected cows from each of the 3 groups. Colostrum was sampled within 12 h *post partum *and on day 3 *post partum *(colostral period). Also a blood sample was taken in association with the first collection of colostrum to determine blood Se level. The Se level in milk and blood was determined twice during the lactation period by random sampling of 6 cows from each group in weeks 6 and 12 of the experiment, respectively (lactation period). Blood samples were collected for biochemical and haematological analysis in week 12 (see below).

### Sampling

During the experimental period, six 250 g samples of grass silage (47.49% dry matter (DM)), hay (88.95% DM), and pasture fodder (22.2% DM) were collected and frozen at -18°C. At the end of the study, feed samples were homogenized, mixed, and the mean Se content on the DM basis was determined.

Blood samples were drawn from the coccygeal vein using the HEMOS^® ^system (1 lithium heparin, 1 K_3_EDTA, and 1 serum tube) (GAMA, Ceske Budejovice, Czech Republic). Milk and colostrum samples were drawn between two calf nursings using a sterile tube. The samples for Se analysis were frozen at -18°C, and analyzed at the end of the study.

### Laboratory analyses

The concentrations of Se in whole blood, milk, and feed were analyzed with hydride generation atomic absorption spectrophotometry (HG-AAS) using the method described by Sturman [16].

The blood was analyzed for urea and total protein content and for the activity of CK and AST in the serum, and for GSH-Px activity in whole blood.

Haematology included erythrocyte count, hemoglobin content and total number of leukocytes. Standard techniques were used for these analyses **(**Central Clinical Laboratories, University of Veterinary and Pharmaceutical Science, CZ). GSH-Px activity was determined by the RANDOX-RANSEL RS 505 kit and the method described by Paglia and Valentine [17].

### Statistical analyses

The data were analyzed by ANOVA test and contrast tests were subsequently used to compare the control group to the other groups and the supplemented groups with each other. One-way ANOVA was used for most of the parameters. The data on colostrum were also analyzed by ANOVA for repeated measure design. For analysis of data on the Se concentration in milk, multi-factorial (2-way) ANOVA was used. The software package Statistica 8.0 (StatSoft, Inc.) was used. Differences were considered as significant when *P *< 0.05.

## Results and discussion

### Analysis of feed

The mean daily intake for the period under study was 14 kg of DM per cow. Roughage consisted of grass silage (0.033 mg Se/kg DM) and hay (0.051 mg Se/kg DM), in a ratio of 1:2 on the DM basis. By the beginning of May, all the cows had calved and were driven to pasture (0.084 mg Se/kg DM). The mean daily intake of the mineral premix was 70 g per head, i.e., 3.5 mg Se per head per day in the supplemented groups. The estimated intake of Se naturally present in the feed was 0.65 mg per head per day for all groups.

### Selenium status of the herd

The average whole blood Se content before experimental Se supplementation was 38.5 μg/L (S.D. 8.4). The blood Se concentration in the control animals remained at this level throughout the study. All animals were considered to be Se deficient at the beginning of the study and the control animals remained deficient throughout the study as levels below 70 μg/L are subnormal [14].

### Colostral period

The animals of the supplemented groups had significantly higher blood Se concentrations at day 1 *post partum *than the controls (*P *< 0.01) (Table 1). Furthermore, the SY group had a higher Se level than the SS animals (*P *< 0.01). These findings correspond to previously published results [7,9]. Weiss [15] compared the results of 10 studies and reported 18% as the median difference in Se concentrations in the blood of animals given organic Se compared to those given inorganic Se. In the present study, the difference observed was greater (36%). This may be explained by breed differences (dairy *vs. *beef), that the cows included in this study were Se deficient at the beginning of the study, and/or that the dose of Se used was higher than in other studies.

**Table 1:** Mean selenium concentrations (μg/L) in whole blood and colostrum of beef cows receiving selenium-enriched yeast (SY), sodium selenite (SS) or no selenium supplementation (C): Analyses performed on day 1 *post partum *(blood and colostrum) and day 3 *post partum *(colostrum).

Group		SY (N = 6)		SS (N = 6)		C (N = 6)	
		x	S.D.	x	S.D.	x	S.D.
Blood		93.0^a^	9.2	68.0^b^	9.8	35.1^c^	3.5
Colostrum	Day 1	72.9^a1^	7.5	56.0^b1^	8.0	27.3^c1^	2.7
	Day 3	47.5^a2^	8.8	18.8^b2^	1.0	10.5^c2^	1.2

^abc ^Values designated by different superscripts within a row differ at a significance level of *P *< 0.05. (Repeated measure analysis for colostrum values – *P *< 0.01).

^1,2 ^Selenium concentration values designated by different superscripts within a column differ at a significance level of *P *< 0.01.

Despite a low blood Se concentration, a relatively high Se content in colostrum just after calving was found in the control group (27.3 μg/L) (Table 1). Interpretation of this finding is difficult due to the lack of corresponding studies. Micetic-Turk *et al. *[6] found a similar level (29.9 μg/L) in colostrum of dairy cattle, but with a corresponding blood concentration of 62 μg/L. Our data are more similar to those found in Belgian Blue cattle [18] showing a very close correlation between colostral and estimated whole blood Se content. It is possible that Se concentrations in colostrum are higher in beef breeds than in dairy breeds due to a lower colostrum production. Similar findings have been made in sheep [19], swine [20], and humans [21], which also produce colostrum in relatively small volumes.

Both the supplemented groups showed higher mean colostrum Se concentrations than the control with the SY group being the highest (Table 1). These findings correspond to previous observations [10,18]. The colostrum Se concentration remains higher even if the animals are given a diet with a twice as high inorganic Se content as organic Se [10].

The colostral Se concentration was significantly reduced on day 3 *post partum *for all groups (Table 1). In the control group, Se concentration dropped to nearly the level present in milk later in the study period (Table 2). In the SY group, however, the colostrum Se concentration on day 3 *post partum *was only 35% lower than that measured just after calving, whereas the SS and control groups showed a 67% and 62% decrease, respectively. The higher Se concentration in the SY group may reflect a higher bioavailability of organic Se. It is possible, that the decrease of Se concentration in all groups is associated with a decreased colostral protein content reflecting the conversion of colostrum into milk as most Se in milk is bound in complexes with proteins [22]. The high Se concentration may also reflect an active transport of Se into the colostrum.

**Table 2:** Mean concentrations of selenium (μg/L) in whole blood and milk of beef cows receiving selenium-enriched yeast (SY), sodium selenite (SS) or no selenium supplementation (C): Analyses performed in weeks 6 and 12 of selenium supplementation.

Group		SY		SS		C	
		x	S.D.	x	S.D.	X	S.D.
		
Week 6 (N = 6)	Blood	89.6^a^	9.1	63.6^b^	7.2	44.4^c^	4.4
	Milk	20.4^a^	2.1	8.3^b1^	1.2	6.9^c^	1.0

Week 12 (N = 6)	Blood	88.6^a^	14.9	65.0^b^	9.5	42.4^c^	11.2
	Milk	19.6^a^	1.2	11.9^b2^	2.2	6.6^c^	1.0

^abc ^Values designated by different superscripts within a row differ at a significance level of *P *< 0.01.

^1,2 ^Milk selenium concentration values designated by different superscripts within a column differ at a significance level of *P *< 0.05

The Se content in colostrum of individual cows is shown in additional file 1 (Individual data on cows analysed for selenium content in colostrum).

### Lactation period

The blood Se concentrations found at weeks 6 or 12 of Se supplementation (Table 2) did not differ from the values found during the colostral period. Analyses of milk Se concentrations did not demonstrate differences between the 2 sampling periods for the SY and control groups, while the Se level increased significantly for the SS group (Table 2). This finding does not correspond to the findings made by Ortman and Pehrson [3], who demonstrated stable Se levels in the milk one week after supplementation with SS. However, the discrepancy might be explained by a more appropriate Se status at the start of their experiment (90 μg Se/L blood).

The milk Se concentration differed significantly among the groups for both weeks 6 and 12 with the SY group having the highest level, the SS group having an intermediate concentration and the control group having the lowest values (Table 2). Similar finding have been found in other studies as reviewed by Weiss [15]. However the difference between the SY and SS groups was rather great. The relative increase in milk Se concentration (compared to the control) was 2.45 and 1.65 times higher in the SY group than in the SS group in weeks 6 and 12, respectively.

The milk Se values of the supplemented groups were rather low compared to those found in other studies [3,9]. However, these studies also showed substantially higher Se content in cows' blood (>100 μg/L). On the other hand, Pehrson *et al. *[7] found very similar milk Se concentrations (SS 12.7 μg/L; SY 17.25 μg/L) but with high Se blood levels (around 125 μg/L). The cause of these discrepancies is not clear.

Data on the Se content in milk of individual animals is shown in additional file 2 (Individual data on cows analysed for selenium content in milk).

### Blood chemistry and haematology (Lactation period)

The biochemical and haematological parameters were not affected by the Se supplementation except for blood urea and GSH-Px (Table 3). The SS group showed an increase in the blood urea level. Different blood urea values between animals supplemented with different Se sources has been reported previously [9], but the findings are contradictory. The findings are not considered to be of biological significance.

**Table 3:** Mean values of blood chemistry and haematology for beef cows receiving selenium-enriched yeast (SY), sodium selenite (SS) or no selenium supplementation (C) for 12 weeks.

	Group			
	SY	SS	C	SEM^1^
	
Analysis				
	
Red blood cells, × 10^12^/L	7.15	6.3	6.03	0.58
White blood cells, × 10^9^/L	7.28	6.47	7.60	0.58
Haemoglobin, g/L	102.0	102.5	99.8	1.43
Total proteins, g/L	68.0	83.2	76.8	7.63
Urea mmol/L	3.20^b^	5.71^a^	3.88^b^	1.29
Creatine kinase, μkat/L	4.39	4.14	4.77	0.72
Aspartate aminotransferase, μkat/L	1.61	1.58	1.66	0.04
Glutathione peroxidase μkat/L*	364.70^a^	283.82^b^	187.46^c^	88.73

^1^Standard error of the mean of 18 cows. 17 degrees of freedom.

^abc ^Statistically significant differences (*P *< 0.05) of urea concentration and glutathione peroxidase activity in SY, SS and C in the same row are designated by different superscripts.

* Glutathione peroxidase activity values were determined in heparinized whole blood

Significant differences in GSH-Px activity were found among all the groups. The SY group showed the highest activity, the SS group an intermediate activity and the control group had the lowest activity (Table 3). However, despite having received Se supplementation for 12 weeks, none of the groups had GSH-Px activity above the minimal reference value (600 μkat/L) [14]. This is surprising since the Se levels in the blood had reached normal levels weeks before, at least for the SY group (Tables 1 and 2).

The relationship between Se saturation and GSH-Px activity has recently been discussed [13]. It is possible that incorporation of Se into the erythrocyte GSH-Px was delayed by the previous Se deficiency thus causing the reduced GSH-Px activity.

The significant difference between the SY and SS groups is partially inconsistent with previous findings as reviewed by Weiss [15], who compared more than 10 studies on this subject. In eight studies, no significant differences in the GSH-Px activity were found in animals supplemented with either organic or inorganic Se. The findings in the present study might be associated with the initial Se deficiency and a more efficient utilization of organic Se.

## Conclusion

Selenium-enriched yeast was much more effective than sodium selenite in increasing the concentration of Se in blood, colostrum and milk, as well as the GSH-Px activity. Cows fed selenium-enriched yeast also showed a slower decrease in colostral Se levels. The findings indicate a higher bioavailability of organic Se. This is possibly more pronounced when treating Se deficient animals. As milk is the only nutritional source of neonatal calves and later a supplementary source of nutrition, the use of organic Se may be of clinical significance.

## Competing interests

The author(s) declare that they have no competing interests.

## Authors' contributions

PS, MB and JH carried out the clinical trial and collected samples of blood, colostrum and milk. JI participated in the design of the study, supervised and participated in data collection. RR carried out the statistical analysis. PS wrote the manuscript. FJ coordinated the work. All the authors have read and approved the final manuscript.

## Supplementary Material

Additional file 1 Individual data on cows analysed for selenium (Se) content in colostrum. SY: Cows receiving organic dietary Se supplementation, SS: Cows receiving inorganic dietary Se supplementation and C: Cows not receiving additional Se. Click here for file

Additional file 2 Individual data on cows analysed for selenium (Se) content in milk. • SY: Cows receiving organic dietary Se supplementation, SS: Cows receiving inorganic dietary Se supplementation and C: Cows not receiving additional Se. Click here for file
