# Supplementary material for: Influence of organic versus inorganic dietary selenium supplementation on the concentration of selenium in colostrum, milk and blood of beef cows
Source: Acta Vet Scand. 2008 Nov 3;50(1):43. doi: 10.1186/1751-0147-50-43 (PMC2585566; doi:10.1186/1751-0147-50-43)
Supplement: Additional file 1 — Individual data on cows analysed for selenium (Se) content in colostrum. SY: Cows receiving organic dietary Se supplementation, SS: Cows receiving inorganic dietary Se supplementation and C: Cows not receiving additional Se. [file 1751-0147-50-43-S1.doc]

Additional file 1.

Individual data on cows analysed for selenium (Se) content in colostrum. SY: Cows receiving organic dietary Se supplementation, SS: Cows receiving inorganic dietary Se supplementation and C: Cows not receiving additional Se.

| Group | Animal identification | Period of Se supplementation prior to calving (weeks) | First sampling | | Second sampling | |
| --- | --- | --- | --- | --- | --- | --- |
|  |  |  | Days after calving | Se content in colostrum | Days after calving | Se content in colostrum |
| SY | SY1 | 6 | 0 | 78.7 | 3 | 58.92 |
| SY | SY2 | 8 | 0 | 64.89 | 3 | 38.6 |
| SY | SY3 | 7 | 0 | 85.48 | 3 | 47.86 |
| SY | SY4 | 9 | 0 | 63.88 | 3 | 59.4 |
| SY | SY5 | 6 | 0 | 72.72 | 3 | 40.5 |
| SY | SY6 | 7 | 0 | 71.46 | 3 | 39.7 |

| Group | Animal identification | Period of Se supplementation prior to calving (weeks) | First sampling | | Second sampling | |
| --- | --- | --- | --- | --- | --- | --- |
|  |  |  | Days after calving | Se content in colostrum | Days after calving | Se content in colostrum |
| SS | SS1 | 6 | 0 | 65.42 | 3 | 20.4 |
| SS | SS2 | 10 | 0 | 48.28 | 3 | 18.58 |
| SS | SS3 | 8 | 0 | 61.27 | 3 | 19.5 |
| SS | SS4 | 6 | 0 | 44.68 | 3 | 17.43 |
| SS | SS5 | 9 | 0 | 52.2 | 3 | 19.2 |
| SS | SS6 | 7 | 0 | 64.41 | 3 | 17.81 |

| Group | Animal identification | Period of Se supplementation prior to calving (weeks) | First sampling | | Second sampling | |
| --- | --- | --- | --- | --- | --- | --- |
|  |  |  | Days after calving | Se content in colostrum | Days after calving | Se content in colostrum |
| C | C1 | 9 | 0 | 30.56 | 3 | 10.4 |
| C | C2 | 8 | 0 | 25.68 | 3 | 8.7 |
| C | C3 | 6 | 0 | 24.64 | 3 | 9.84 |
| C | C4 | 7 | 0 | 30.71 | 3 | 12.45 |
| C | C5 | 7 | 0 | 28.42 | 3 | 10.16 |
| C | C6 | 10 | 0 | 23.91 | 3 | 11.7 |
