# Supplementary material for: Influence of organic versus inorganic dietary selenium supplementation on the concentration of selenium in colostrum, milk and blood of beef cows
Source: Acta Vet Scand. 2008 Nov 3;50(1):43. doi: 10.1186/1751-0147-50-43 (PMC2585566; doi:10.1186/1751-0147-50-43)
Supplement: Additional file 2 — Individual data on cows analysed for selenium (Se) content in milk. • SY: Cows receiving organic dietary Se supplementation, SS: Cows receiving inorganic dietary Se supplementation and C: Cows not receiving additional Se. [file 1751-0147-50-43-S2.doc]

Additional file 2.

Individual data on cows analysed for selenium (Se) content in milk. SY: Cows receiving organic dietary Se supplementation, SS: Cows receiving inorganic dietary Se supplementation and C: Cows not receiving additional Se.

| Group | First sampling | | | | Second sampling | | | |
| --- | --- | --- | --- | --- | --- | --- | --- | --- |
|  | Animal identification | Period of Se supplementation prior to sampling (weeks) | Days after calving | Se content in milk | Animal identification | Period of Se supplementation prior to sampling (weeks) | Days after calving | Se content in milk |
| SY | SY7 | 6 | 10 | 21.43 | SY10 | 12 | 54 | 20.74 |
| SY | SY8 | 6 | 14 | 16.61 | SY13 | 12 | 32 | 18.56 |
| SY | SY9 | 6 | 16 | 19.73 | SY14 | 12 | 14 | 21.43 |
| SY | SY10 | 6 | 12 | 23.54 | SY15 | 12 | 40 | 19.73 |
| SY | SY11 | 6 | 18 | 21.65 | SY16 | 12 | 19 | 17.75 |
| SY | SY12 | 6 | 13 | 19.65 | SY17 | 12 | 38 | 19.68 |

| Group | First sampling | | | | Second sampling | | | |
| --- | --- | --- | --- | --- | --- | --- | --- | --- |
|  | Animal identification | Period of Se supplementation prior to sampling (weeks) | Days after calving | Se content in milk | Animal identification | Period of Se supplementation prior to sampling (weeks) | Days after calving | Se content in milk |
| SS | SS7 | 6 | 10 | 9.1 | SS12 | 12 | 58 | 9.14 |
| SS | SS8 | 6 | 14 | 9.75 | SS13 | 12 | 32 | 14.36 |
| SS | SS9 | 6 | 17 | 9.57 | SS11 | 12 | 53 | 13.63 |
| SS | SS10 | 6 | 13 | 6.79 | SS14 | 12 | 24 | 9.33 |
| SS | SS11 | 6 | 11 | 7.82 | SS15 | 12 | 18 | 14.3 |
| SS | SS12 | 6 | 16 | 7.04 | SS16 | 12 | 35 | 10.85 |

| Group | First sampling | | | | Second sampling | | | |
| --- | --- | --- | --- | --- | --- | --- | --- | --- |
|  | Animal identification | Period of Se supplementation prior to sampling (weeks) | Days after calving | Se content in milk | Animal identification | Period of Se supplementation prior to sampling (weeks) | Days after calving | Se content in milk |
| C | C7 | 6 | 19 | 6.69 | C1 | 12 | 28 | 8.1 |
| C | C8 | 6 | 12 | 8.04 | C10 | 12 | 55 | 7.61 |
| C | C9 | 6 | 16 | 7.81 | C13 | 12 | 24 | 6.32 |
| C | C10 | 6 | 13 | 7.52 | C14 | 12 | 16 | 5.91 |
| C | C11 | 6 | 10 | 6.58 | C15 | 12 | 35 | 5.01 |
| C | C12 | 6 | 18 | 5.01 | C16 | 12 | 14 | 6.87 |
